# Profile of hospitalizations due to otorhinolaryngologic morbidity requiring surgical treatment. Brazil, 2003

**DOI:** 10.1016/S1808-8694(15)31235-0

**Published:** 2015-10-20

**Authors:** Mariana de Carvalho Leal Gouveia, Fábio José Delgado Lessa, Mirella Bezerra Rodrigues, Silvio da Silva Caldas Neto

**Affiliations:** Ph.D. in Science (Otorhinolaryngology), FMUSP, Deputy Professor, Department of Surgery, UFPE; Ph.D. studies in Nutrition under course, UFPE. Assistant Professor, Department of Surgery - UFPE; Master studies in Public Health under course, Centro de Pesquisa Aggeu Magalhães - Fundação Oswaldo Cruz (CPqAM/Fio Cruz), Speech and Language Therapist; Full Professor, Joint Professor of Otorhinolaryngology, Federal University of Pernambuco - UFPE. Centro de Pesquisas Aggeu Magalhães - Fundação Oswaldo Cruz (CPqAM/FIO CRUZ)

**Keywords:** epidemiology, surgical procedures, otorrhinolaringology

## Abstract

**Aim:**

To analyze the profile of the surgical procedures related to otorhinolaryngology in Brazil in the year 2003.

**Study Design:**

This was an observational, descriptive, cross-sectional study.

**Methods:**

We analyzed 80,030 surgical procedures performed in 27 Brazilian States during the period from January to December 2003. The data were obtained from the Hospital Information System of the Ministry of Health. The inclusion factor was a surgical procedure in otorhinolaryngology (ORL). All files were processed with the TABWIN software.

**Results:**

In 2003, 80,030 ORL-related surgical procedures were performed in Brazil. The Southeast region had the largest number of procedures (53.08%), followed by the South and Northeast regions (19.6% and 15.6%, respectively). Regarding the group of procedures, surgeries of the pharynx represented 45% of ORL procedures. Procedures of high complexity were more numerous in the ear surgery group. Regarding the distribution of the type of attending institution, the highest concentration of surgical procedures occurred in philanthropic hospitals, followed by state and university public hospitals. The table adopted by SUS for payment of ORL surgical procedures has not been updated for the procedures currently performed, with the consequent inappropriate notification of some types of surgery.

**Conclusion:**

Knowledge about the profile of surgical hospitalizations related to ORL permits the identification of the peculiarities of the different regions that can help health-managing authorities to make decisions in order to guarantee the principles recommended by SUS regarding access to health services.

## INTRODUCTION

Otorhinolaryngology (ENT) is a clinical surgical specialty that has grown a lot in Brazil for the past years. However, public healthcare for this specialty has presented major disproportions in different regions of Brazil.

The small number of centers that train specialists in Otorhinolaryngology in some regions, such as the North and Northeast regions and the main concentration in the Southeast region makes it one of the main determining factors for the uneven distribution of services, together with financial factors, considering that it is a specialty that requires high cost equipment.

The Brazilian Constitution of 1988 has incorporated changes to the role of the government and deeply changed the legal-institutional framework of the public healthcare service, creating new relations between the different governmental levels, new roles to players in the area and has given rise to the Universal Healthcare System (SUS - Sistema Único de Saúde)^1^.

According to the Ministry of Health, the doctrine of SUS includes the following factors: universality, based on assurance of healthcare to any and all citizens, equity, ensuring actions and services of all levels according to the complexity required in each case, and integrity ensuring that citizens have access to health promotion, protection and rehabilitation actions, creating a system capable of providing integral healthcare 2. Medical services, therefore, should be organized so as to ensure compliance with these principles.

The repercussions of pathologies related with Otorhinolaryngologists can be recognized in many different areas, such as occupational health, such as voice professionals and work-related laryngopathies 3, which may lead to inability to work in the role and consequent transfer of function. Another important aspect is hearing impairment: it is estimated that in the Brazilian population 1.5%, that is, about 2,250,000 inhabitants have hearing loss, ranked third among all impairments in the country 4.

So that we can develop actions and intervention strategies directed to medical care improvement it is necessary to have epidemiological profile of the affections and interventions and regional distribution.

The Health Information System (SIS) performs a fundamental role in Epidemiology in Health Care, aiming at making the diagnosis of healthcare status and assessment of public policy impact 5.

The Hospital Information System SUS (SIH/SUS) comprises an administrative database. Despite the fact that the main purpose of the system was hospitalization compensation sponsored by SUS, it has enabled the generation of important information related to morbidity such as the description of care and resources used, despite the limitations 6, 7. By means of studies carried out in such systems, we have considered the use of this database that may represent an important advance in the assessment actions and quality improvement of healthcare 5.

In ENT, this type of studies is scarce, and it is necessary to get to know better the profile of hospital procedure in ENT in Brazil and their implications concerning better planning and distribution of such actions.

Taking these considerations into account, we intended to study and analyze the hospitalization profile by ENT morbidity with surgical treatment in Brazil in 2003, characterizing ENT surgical procedures according to state, number of procedures by hospital, complexity, nature of hospital, age, gender and identification of mean length of stay for surgical procedures, specifying the value of professional, hospital and diagnostic support services.

## MATERIAL AND METHOD

We analyzed 80,030 surgical procedures within a total of 1,779 SUS-associated hospitals that have surgical hospitalization in ENT in 27 states of Brazil, within January and December 2003.

It was an observation descriptive cross-section study.

The studied surgical procedures were analyzed by group of procedures related with ENT, divided by anatomical region (ear, pharynx, larynx, nose).

The other analyzed variables were: region, state, complexity (high and medium complexity), month of competence (previous month of submission of bill, which is normally the discharge month), type of provider (legal nature of provider classified as federal, state, municipal, non-profitable and university), mean value of hospital services (SH), professional services (SP), complementary tests for diagnosis and therapy (SADT), for the bills paid.

The analyzed data were collected from the files of hospital production RDUFAAMM.DBC in the period from January to December 2003, kept by the Hospital Information System, Ministry of Health. The inclusion factor was ENT surgical procedure. All files were processed by the software TABWIN developed by the Department of Information Technology, Ministry of Health.

Given that they were data available on the Internet and without identification and because they were aggregated by Healthcare center and municipalities, there was no need for Informed Consent Term and approval of the Ethics Committee, and the source where they were collected from was: Sistema de Informação Hospitalar/Ministério da Saúde.

## RESULTS

In Brazil, in 2003, 80,030 surgical procedures in ENT-related areas were performed. Table 1, Table 2 show the distribution of these procedures in the 5 regions of the country and the respective states.

**Table 1:** Distribution of surgical procedures in Otorhinolaryngology by region and state. Brazil, 2003.

Region and state	N	%
**North Region**	**3745**	**4,68**
Rondônia	208	0,26
Acre	386	0,48
Amazonas	326	0,41
Roraima	37	0,05
Pará	2317	2,90
Amapá	55	0,07
Tocantins	416	0,52
**Northeast Region**	**12768**	**15,95**
Maranhão	407	0,51
Piauí	588	0,73
Ceará	2329	2,91
Rio Grande do North	1250	1,56
Paraíba	1302	1,63
Pernambuco	1917	2,40
Alagoas	603	0,75
Sergipe	720	0,90
Bahia	3652	4,56
**Southeast Region**	**42476**	**53,08**
Minas Gerais	7901	9,87
Espírito Santo	1759	2,20
Rio de Janeiro	4148	5,18
Sao Paulo	28668	35,82
**South Region**	**15676**	**19,59**
Paraná	7704	9,63
Santa Catarina	2652	3,31
Rio Grande do Sul	5320	6,65
**Center West Region**	**5365**	**6,70**
Mato Grosso do Sul	450	0,56
Mato Grosso	804	1,00
Goiás	2743	3,43
Distrito Federal	1368	1,71
**Total**	**80.030**	**100,00**

**Table 2:** Distribution of surgical procedures in Otorhinolaryngology by region. Brazil, 2003.

Procedure group	North		Northeast		Southeast		South		Center West		Total
	N	%	N	%	N	%	N	%	N	%	
Ear surgery	306	8,2	1559	12,2	6652	15,7	1977	12,6	703	13,1	11197
Nose surgery	1045	27,9	4121	32,3	11870	27,9	3837	24,5	1523	28,4	22396
Pharynx surgery	1844	49,2	4553	35,7	19032	44,8	8089	51,6	2600	48,5	36118
Larynx surgery	550	14,7	2535	19,9	4922	11,6	1773	11,3	539	10,0	10319
**Total**	**3745**	***100,0***	**12768**	***100,0***	**42476**	***100,0***	**15676**	***100,0***	**5365**	***100,0***	**80.030**

The distribution of the surgical procedures according to complexity is shown in Table 3. We can see that only 1.56% (n = 1,249) of the procedures performed in 2003 were of high complexity. Table 4 presents a classification of hospitals according to provider by region.

**Table 3:** Distribution of surgical procedures in Otorhinolaryngology by procedure complexity. Brazil, 2003.

Procedure group	High Complexity		Medium Complexity		Total
	N %		N %		
Ear surgery	640	5,71	10557	9,28	11197
Nose surgery	577	2,57	21819	97,43	22396
Pharynx surgery	32	0,08	36086	99,92	36522
Larynx surgery	0	0	10319	100	10319
Total	1249	1,56	78781	98,44	80.030

**Table 4:** Distribution of surgical procedures in Otorhinolaryngology by type of provider and region. Brazil, 2003.

Region and state	North	Northeast	Southeast	South	Center West	Total
Hired	1113	2150	2866	2049	676	8854
Federal	81	570	1465	981	650	3747
State	581	2407	9291	1718	707	14704
Municipal	254	641	3201	791	337	5224
Non-profitable	1581	4247	19227	6543	1679	33277
University	135	2753	6426	3594	1316	14224
**Total**	**3745**	**12768**	**42476**	**15676**	**5365**	**80.030**

Surgeries were performed 55.18% (n = 44,163) of the times in male patients and 44.81% (n = 35,867) in female patients (Table 5). The distribution of surgical procedures by age range is shown in Table 6.

**Table 5:** Distribution of procedures in Otorhinolaryngology by gender. Brazil, 2003.

Procedure group	Male	%	Female	%	Total
Ear surgery	5200	48,56	5507	51,44	10707
Nose surgery	13609	60,0	8787	40,0	22396
Pharynx surgery	19243	52,56	17365	47,43	36608
Larynx surgery	6111	59,22	4208	40,77	10319
**Total**	**44163**	***55,18***	**35867**	***44,81***	**80.030**

**Table 6:** Distribution of procedures in Otorhinolaryngology by age range. Brazil, 2003.

Procedure group	≤ 4 years	5–9y	10–19y	20–39y	40–59y	≥ 60 y	Total
Ear surgery	762	1502	2880	3858	1865	330	11197
Nose surgery	278	614	5268	11419	3758	1059	22396
Pharynx surgery	9403	15692	7004	3001	778	240	36118
Larynx surgery	1267	1230	1140	1766	2946	1970	10319
**Total**	**11710**	**19038**	**16292**	**20044**	**9347**	**3599**	**80.030**

Values referring to cost with hospitalization, hospital services, professional service (including the whole team: surgeon, assistant surgeon and anesthesiologist), and complementary diagnostic and therapeutic services are shown in Table 7.

**Table 7:** Description of mean value of hospitalization, hospital services, professional and complementary tests by ENT surgical procedure. Brazil, 2003.

Frequency	Frequency	Mean value	Mean Hospital Service Value	Mean Professional Service Value	Mean Complementary Test Value (SADT)
Tonsillectomy and/or					
Adenoidectomy	33377	452,5	202,7	167,1	12,5
Peritonsillar abscess drainage	298	161,96	82,49	62,48	3,00
Pharyngeal abscess drainage	221	247,36	143,24	65,42	10,15
Pharynx tumor exeresis	261	312,51	155,13	101,67	14,36
Paranasal sinus tumor exeresis	217	282,78	130,67	101,17	10,64
Thyroglossus cyst exeresis	3052	212,04	97,05	77,76	5,13
Brachial cyst exeresis	1472	288,85	138,76	100,52	8,79
Papilloma exeresis and bronchoscopy	376	307,78	126,42	130,81	10,57
Tympanoplasty (type I unilateral)	2701	297,29	156,25	102,37	1,16
Tympanoplasty (type I - bilateral)	377	474,15	134,59	240,71	4,61
Tympanoplasty (other types unilateral)	2497	384,97	132,35	176,04	4,42
Otological microsurgery	1818	298,51	156,41	101,39	1,21
Radical mastoidectomy	1530	400,65	143,56	178,26	5,63
Subtotal mastoidectomy	588	285,92	143,72	99,11	2,40
Stapedectomy	713	402,07	134,96	185,84	4,54
Otological microsurgery in patients with deformities	350	449,78	200,86	173,66	1,68
Cochlear implants	150	50632,20	943,75	458,99	120,55
Laryngoscopy with papilloma exeresis	4374	179,68	78,91	67,94	5,10
Direct laryngoscopy for foreign body removal	1275	190,08	81,01	74,93	5,56
Partial laryngectomy	529	618,78	344,50	167,42	33,37
Turbinectomy	2455	213,54	104,66	76,87	2,80
Septoplasty (septum deviation)	4901	183,20	102,88	55,77	2,75
Non-cosmetic repair septoplasty	694	194,22	123,87	48,52	1,92
Surgical reduction of nose bones	6465	215,68	122,12	64,60	3,76
Non-cosmetic repair rhinoplasty	2601	210,67	136,70	52,95	2,32
Rhinoplasty in cleft lip-palate patients	326	401,61	247,99	103,94	3,96
Rhinoplasty for post-traumatic effects	825	206,26	136,38	49,55	2,19
Partial nose reconstruction	902	246,95	140,56	77,36	3,22
Total nose reconstruction	278	260,79	152,29	76,22	4,22
Ethmoid sinusotomy	714	280,84	149,91	74,36	10,72
Maxillary sinusotomy	892	183,57	102,03	53,62	4,88
Transmaxillary sinusotomy	548	223,22	145,34	49,53	3,58
Frontal sinusotomy	159	210,31	135,72	50,83	3,41
Others	2094	575,94	318,75	153,28	30,63
**Total**	**80.030**	**347,39**	**122,46**	**90,16**	**6,14**

SADT: complementary tests for diagnosis and therapy

## DISCUSSION

Social-economic differences that exist in Brazil are clearly reflected in many aspects, which also apply to medical care. According to the obtained results, the Southeast region is the one that performs the most procedures (53.08%), followed by the South and Northeast regions, with 19.6% and 15.6%, respectively. As to states, Sao Paulo concentrates 35% of procedures in Brazil, followed by Minas Gerais with 9.9%. The North region is the region that has the smallest number of surgical procedures in the country. In the Northeast, the state of Bahia presented 4.6% of procedures, followed by Ceara and Pernambuco with 2.9 and 2.4, respectively. In our opinion, this distribution is a result of the fact that the Southeast region, especially the city of Sao Paulo, in addition to being highly populated 8, it is the largest medical center in the country, concentrating many hospitals and medical centers. However, such data show the uneven distribution of ENT medical care in the different regions of the country, probably a reflex of the uneven economic heterogeneous distribution and development among the regions.

When we analyze it by group of procedure (Table 2), pharyngeal surgeries represent 45% of ENT procedures, which is owed to the influence of tonsillectomy and adenoidectomy, which are the most frequent ENT surgeries in Brazil and also in the USA 9. In all regions, this is the group of procedures most frequently performed. It is important to point out the fact that ear surgeries represent 8% of procedures in the North and over 12% in the other regions, up to 15.7% in the Southeast, which include high complexity surgeries whose surgical material is not necessarily available in SUS centers in other states, which results in some patients being referred to outside treatment, sometimes in other regions of the country, generating higher costs for the resolution of the case.

The complexity of a surgical procedure is classified by SUS into high and medium complexity. Medium complexity comprises a set of actions and ambulatory and hospital services that aim at dealing with the main healthcare problems of the population, whose clinical practice demands the availability of specialized professionals and the use of diagnostic and therapeutic technological support, which does not justify its provision in other cities of the country. High complexity, in turn, is high technology, high quality and high cost services. In Table 3 we can see the distribution of these ENT surgical procedures according to complexity.

High complexity ENT procedures comprise cochlear implant and surgeries in patients with craniofacial deformities (pharynx, nose and ear) that are part of SIPAC (High Complexity Procedure Information System). The group of ear surgery comprises most of these procedures, maybe because of the presence of cochlear implant, which has been expanding in Brazil. In 2003, out of 640 high complexity procedures, 150 were cochlear implants (SIH/SUS, 2004). It is a procedure that started to be performed by SUS in 1993 and up to 2003 three states (Rio Grande do Norte, Rio Grande do Sul and Sao Paulo) had performed the procedure, reflecting the need to be expanded to other centers to provide the universal action proposed by SUS, so that the whole population can have access to healthcare services for integral healthcare provision.

As to distribution of ENT procedures according to type of provider, we observed that there is higher concentration of surgical procedures performed in non-profitable hospitals, followed by state and university centers (Table 4). We also observed that contracted hospitals (private SUS-related hospitals) amounted to only 11% of these procedures performed in the country. The North region presented higher productivity of this type of provider compared to the others, with 29% surgical procedures, and the Southeast region is the one that has fewer surgeries performed in contracted hospitals (6.74%). Our opinion is that owing to the fact that the fees paid for ENT procedures are low compared to other surgical procedures performed by other specialties, in the private sector, SUS-related entities do not feel encouraged to perform these procedures and, at the same time, high complexity procedures that are well compensated have high level of requirements, hindering the accreditation of this type of provider.

The low production of federal hospitals is justified by the small number of this type of hospital per state. We have to take into account the fact that university hospitals, differently from other type of providers, do not intend to have high yield, because they have their activities directed to teaching and research.

As to analysis and distribution by gender (Table 5), we detected slight predominance of male gender in ENT procedures in general. We observed similar distribution concerning gender in all procedure groups, which was expected given that we analyze many types of surgery in the same group. Knowing that most surgical pathologies in ENT do not have different distribution concerning gender, except for few such as otosclerosis, which is more frequent in women 10, and larynx cancer, which is more frequent in male subjects 11.

As to age range distribution (Table 6), pharynx surgeries, especially tonsillectomy with and without adenoidectomy, were more frequent in school-age children (5–9 years) and pre-school age children (≤ 4 years), which was already expected, given that the lymphoid tissue reaches maximum growth between the age of 4 and 10 years, period of maximum clinical manifestation leading to surgical procedure 12. Ear surgeries were more frequent in young adult age, which was already expected given that most surgical pathologies in this group result from chronic inflammatory processes of slow progression. Laryngeal surgeries were more prevalent in adults (40 to 59 years) and the elderly (≥ 60 years). In this group, we can include pathologies such as laryngeal neoplasm that affects the population with mean age of 58 years, according to the national study carried out by Steffen and Corrêa (1994)^10^, and laryngeal benign pathologies, especially those resulting from speech trauma that may be related to some specific professional activities more prevalent in older stages of life, reaching the economically active population.

We observed that some procedures shown in Table 7 described as laryngectomy, nose reconstruction, rhinoplasty may be performed also by professionals of related areas such as head and neck surgeon and plastic surgeon. This difficulty to select more restrictively procedures performed by Otorhinolaryngologists is owed to the fact that SIH does not identify which professional performed the procedure, even though there is a procedure table that includes the description of which professionals are trained to perform each procedure. We decided to select by group of procedure (ear, nose, pharynx and larynx) because they are areas of competence of Otorhinolaryngology, because there is no selection by performing professional, even though we are aware that some of the procedures could have been performed by some other specialist.

We can also observe that in the surgical procedure table in force some procedures that have been recently implemented as a result of surgical and technological advance have not been included yet, such as the case of nasal endoscopic surgeries applied specially in the surgical approaches of sinusal surgeries, which is well recognized as a safe technique that has more benefits to the patients than the conventional technique 13. This type of surgery demands higher expenses for its execution because of surgical material used and videoendoscopic equipment, and as we could observe there is no differentiation in terms of payment for endoscopic or conventional procedures, which may lead to situations in which the hospitals that do not have other source of income can not purchase such special type of material. The procedures that are paid higher mean values are high complexity procedures such as cochlear implant and craniofacial deformity surgeries, which involve high cost materials, but the price increase of expensive materials is not necessarily followed by increase in fees paid by SUS.

Considering such factors, in addition to lack of attention when filling out the forms that generate notification of information sources by professionals that perform the procedures, which may imply inappropriate information, we should be very careful when analyzing data generated from such instruments.

## CONCLUSION

The obtained data show uneven distribution concerning ENT surgical care in the regions and states of Brazil. The most relevant aspects were:

• More than 50% of ENT surgical procedures are performed in the Southeast region.
• High complexity procedures represent only 1.56% of surgical procedures in ENT, which is the group that generates the higher number of these procedures is ear surgery, contributing to such data the cochlear implant surgery, but it is still less available in Brazil than its demand.
• Non-profitable hospitals were the ones that performed the most such surgical procedures, and there was little participation of private (hired) sector as a result of low fees paid for such procedures.
• The surgical procedure fee table adopted by SUS is not updated to include the most current procedures, leading to inappropriate notification of some types of surgeries.

By knowing about the profile of surgical hospitalization in Otorhinolaryngology in Brazil, we may identify the particularities of distribution concerning the different regions that may support healthcare managers in making decisions to ensure the principles adopted by SUS in healthcare access.
